# Left Ventricular Mass with Delayed Enhancement as a Predictor of Major Events in Patients with Myocarditis with Preserved Ejection Fraction

**DOI:** 10.3390/jcm11206082

**Published:** 2022-10-14

**Authors:** Nicolò Ghionzoli, Annalaura Gismondi, Giulia Elena Mandoli, Lucia Spera, Alex Di Florio, Flavio D’Ascenzi, Matteo Cameli, Luna Cavigli, Carlotta Sciaccaluga, Salvatore Francesco Carbone, Giovanni Donato Aquaro, Serafina Valente, Marta Focardi

**Affiliations:** 1 Department of Medical Biotechnologies, Division of Cardiology, University of Siena, 53100 Siena, Italy; 2 Unit of Diagnostic Imaging, Department of Medical, Surgical and Neuro Sciences and of Radiological Sciences, University of Siena, Azienda Ospedaliero-Universitaria Senese, Viale Bracci 16, 53100 Siena, Italy; 3 Fondazione Toscana Gabriele Monasterio, Via Moruzzi 1, 56124 Pisa, Italy

**Keywords:** acute myocarditis, cardiac magnetic resonance, late gadolinium enhancement, myocarditis with preserved ejection fraction

## Abstract

***Background***: Cardiac Magnetic Resonance (CMR) has a key role in subjects presenting with acute myocarditis, independent from left ventricular ejection fraction; it is widely used as a non-invasive imaging test for both diagnostic and prognostic purposes. However, poor data is available about the CMR-derived prognostic parameters of acute myocarditis with preserved ejection fraction (AMpEF). The aim of this study was to investigate the role of CMR in predicting outcomes in patients followed up for AMpEF, using a composite endpoint of all-cause mortality and hospitalization for heart failure (HF). ***Methods***: We retrospectively enrolled 61 patients with diagnosed AMpEF. All patients underwent biohumoral, echocardiographic and CMR evaluation in the acute phase. Myocarditis was confirmed by Lake–Louis criteria assessed on CMR images. Mean follow-up was 4.8 ± 0.6 years during which a composite endpoint of all-cause mortality and hospitalization for HF was investigated. ***Results***: The population was fairly homogeneous regarding baseline clinical features. In particular, no significant differences in age and main cardiovascular risk factors were found between patients with and without events at follow-up. Seven patients met the endpoint. They had significantly higher levels of circulating neutrophils in the acute phase (76 ± 7% vs. 61 ± 11%, *p* = 0.014) and a higher amount of left ventricular mass with delayed enhancement (DE-LVM, 18 (14–29.5) vs. 12 (8–16) g, *p* = 0.028). At Cox univariate analysis, DE-LVM was the only significant predictor of endpoint, regardless of the site of inflammation. ***Conclusions***: DE-LVM can predict the composite endpoint of all-cause mortality and hospitalization for HF in a population of patients with AMpEF, representing a new added tool for prognostic stratification.

## 1. Introduction

According to the World Health Organization and the International Society and Federation of Cardiology, myocarditis is an inflammatory disease of the heart muscle, whose diagnosis is based on histological, immunological and immunohistochemical criteria [1].

Despite its clear anatomopathological findings, myocarditis is often underdiagnosed, especially in the context of a preserved left ventricular ejection fraction. Myocarditis is clinically evident in 8–10 persons out of 100,000 subjects, but its prevalence on autopsy reaches 44 persons out of every 100,000 [2]. This suggests a high rate of asymptomatic patients that leads to underestimating its real incidence, especially in young patients [3]. In this sense, cardiac magnetic resonance (CMR) has changed both the epidemiology and our knowledge about myocarditis thanks to the ability to identify edema and delayed enhancement [4]. It is the gold standard for diagnosis and quantification of disease extension, with an increasing role in stratifying prognosis.

The classification of myocarditis is controversial and still an object of debate. It can present with a wide clinical spectrum, from asymptomatic cases to cardiogenic shock, with variable symptoms according to the extension and the etiology [3]. Etiological, clinical, histological and echocardiographic classifications have been proposed through the years. Etiological classification distinguishes among infectious, immune-mediated and toxic myocarditis; histology identifies lymphocytic, eosinophilic, polymorphic, giant-cell myocarditis and cardiac sarcoidosis; and clinical classification distinguishes fulminant, acute, chronic active and chronic persistent myocarditis [5].

According to echocardiographic parameters, a classification based on left ventricular ejection fraction is often used because of its simple determination, with consequent impact on the therapeutic strategy. Acute myocarditis with preserved ejection fraction (AMpEF) is still a nebulous clinical entity with scarce and often conflicting evidence in the literature, without evidence-based therapeutic tools. Recently, Georgiopoulos et al. also showed that the presence of late gadolinium enhancement carries independent prognostic value, irrespective of LVEF [6]. Other data come from the ITalian multicenter study on Acute MYocarditis (ITAMY) [7], as the authors found that late gadolinium enhancement (LGE) in the mid-wall layer of the anteroseptal segments of patients with AMpEF was associated with more unfavorable outcomes.

**Figure 1:**
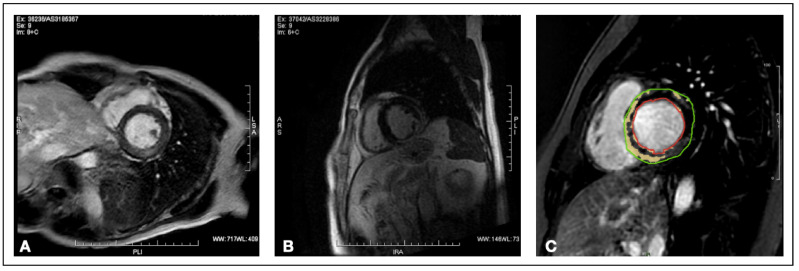
T1-weighted cardiac magnetic resonance short-axis of patients with different degrees of delayed enhancement. (**A**) Patient with mild presence of delayed gadolinium enhancement. (**B**) Patient with diffuse, sub-epicardial and mid-wall localization of delayed gadolinium enhancement. (**C**) Late gadolinium enhancement mass quantification technique. The green circle represents the sub-epicardial layers of myocardium, the red circle represents the sub-endocardial layers.

In particular, to our knowledge, no study has evaluated the amount of left ventricular mass with delayed enhancement (DE-LVM) as a tool for outcome stratification specifically in AMpEF patients (Figure 1). The identification of risk factors for poor outcome may help to stratify patients who need a closer follow-up, as well as to provide effective treatments. With our study, we attempted to identify CMR-related predictors of poor outcome in patients with AMpEF, in particular DE-LVM, using a composite endpoint of all-cause mortality and hospitalization for heart failure (HF).

## 2. Methods

### 2.1. Study Population

We retrospectively analyzed data of patients referred to our Cardiological Department for acute myocarditis from January 2011 to December 2018. In the acute phase, the diagnosis of myocarditis was suspected on the basis of the combination of personal history, symptoms, physical examination, transthoracic echocardiography and bio-humoral assays, and then confirmed by Lake–Louis criteria on CMR images. Ejection fraction was assessed both by transthoracic echocardiography and CMR in each subject, but only patients with preserved LVEF (≥50%) at CMR were included. Exclusion criteria were: LVEF at CMR < 50%, patients aged less than 18 years old, patients who underwent previous heart transplantation/left ventricular assist device implantation, patients with transvenous pacemaker/defibrillator, or acute HF at presentation.

We considered a composite endpoint of all-cause mortality and hospitalization for HF. The study population was then divided into two groups according to occurrence of composite endpoint at follow-up. Informed consent regarding the anonymous use of personal data was obtained from each patient. The study was conducted in accordance with the Declaration of Helsinki [8].

### 2.2. Biohumoral Evaluation

Blood samples were drawn after an overnight fasting period the day CMR was performed. Samples were stored at −80 °C. All patients underwent a thorough biohumoral investigation consisting of complete blood count, serum creatinine, C-reactive protein, hepatic function and high-sensitivity troponin. All the assays were run according to manufacturer instructions. Information about each biomarker was available for all patients.

### 2.3. Transthoracic Echocardiography

Echocardiographic examination was performed at admission, according to the American Society of Echocardiography/European Association of Cardiovascular Imaging recommendations for chamber quantification [9], using the machine Vivid E9 GE Medical System, Northern, Norway.

LVEF, left ventricular and left atrial volume were assessed using the biplane modified Simpson method from the apical 4- and 2-chamber views. Dimensions of left ventricle and left atrium were indexed to body surface area. From the 4-chamber view, tricuspid annulus plane systolic excursion (TAPSE) was measured by M-mode [10]. Systolic pulmonary artery pressure (sPAP) was estimated as the sum of systolic trans-tricuspid pressure gradient and of right atrial pressure derived from the diameter and collapsibility of the inferior vena cava [11].

### 2.4. Cardiac Magnetic Resonance

CMR was performed within three days from hospital admission, using a 1.5 T whole-body scanner (GE MedicalSystems). An eight-channel cardiac phased-array receiver surface coil was used for signal reception. In each patient, a set of contiguous short-axis views was acquired from the tricuspid plane to the apex with a minimum of 30 cineframes for each slice with the following parameters: slice thickness 8 mm, no gap, eight views per segment, NEX 1, field of view 40 cm, phase field of view 1, matrix 224 × 224, reconstruction matrix 256 × 256, flip angle 458, TR/TE 3.5/1.5 and bandwidth 125 kHz. According to the protocols recommended by the Society for Cardiovascular Magnetic Resonance, we acquired cine steady-state free precession (cine-SSFP) images, T_2_-weighted imaging, and LGE at 10 min after gadolinium injection in the short-axis (9 to 13 images covering the entire LV), 2-chamber and 4-chamber planes [12,13,14]. In order to perform segmental analysis, the left ventricle was divided into 17 segments according to the standardized 17-segment model: apical cap, 4 apical, 6 mid-cavity and 6 basal [15]. On T2-weighted images, edema was considered present when the ratio of signal intensity between the myocardium and the mean signal intensity of the skeletal muscle was ≥2. The occurrence of edema was evaluated in each of the 17 LV segments.

### 2.5. Late Gadolinium Enhancement Quantification

Semi-automated quantification was performed as follows: epicardial and endocardial left ventricular (LV) contours were carefully placed manually on all LGE images. The remote non-LGE reference region of each LGE slice was placed adjacent to the region of LGE so that the reference region was at approximately equal distance from the anterior receiver coils. We believe this method minimizes any modifying effect from LGE location on the robustness of the LGE quantification. LGE mass was then quantified by semi-automatic methods using a signal intensity threshold of > 2-SD above a reference region of remote myocardium (adjacent to the region of LGE and approximately equal in distance to anterior receiver coils) in the same slice, and using regions defined as above 50% of maximal signal intensity of the enhanced area for the full width at half maximum approach. Artifacts were manually erased. In all methods, LGE mass (in grams) was then expressed as a percentage of total LV mass determined by balanced steady-state free precession cine images [16].

### 2.6. Follow-Up Evaluation

After inpatient baseline evaluation, patients were followed up at our outpatient clinic. Independent interviewers obtained data from the patients, relatives or general practitioners. Independent physicians performed follow-up resting echocardiography. The follow-up period lasted until May 2020. Information about the time and cause of death was retrieved from death certificates and post mortem reports.

### 2.7. Statistical Methods

Statistical analysis was performed using IBM SPSS 25.0 program (1989–2017, LEAD technologies Inc., Charlotte, NC, USA). The type of distribution of variables was assessed using the Kolmogorov–Smirnov test. Continuous variables with normal distribution are presented as mean ± standard deviation, whereas continuous variables with non-normal distribution as median and interquartile range. Non-continuous variables are presented as frequency and percentage.

Differences among the two groups (patients who met the primary endpoint and those who did not) were assessed using aStudent’s *t*-test for independent samples for what concerns normally-distributed continuous variables; to assess differences in non-normally-distributed continuous variables, a non-parametric Mann–Whitney test was performed; non-continuous variables were assessed using a Chi-square test or Fischer test as appropriate. In each of the above-mentioned cases, two tailed *p* values < 0.05 were considered significant. Survival was assessed with Kaplan–Meier analysis, after dividing our population into patients with left ventricular mass with delayed enhancement (DE-LVM) above and below median value. A Kaplan–Meier curve is presented at 66-month outcome. Differences in survival between groups were tested with a log-rank test (Mantel–Cox).

Univariate Cox analysis was performed in order to identify predictors of the composite endpoint.

## 3. Results

### 3.1. Baseline Features

A thorough descriptive analysis of baseline clinical, biohumoral and instrumental features is presented in Table 1. We enrolled a total population of 61 AMpEF patients with a mean age of 39 ± 12 years, 80% males. The overall prevalence of hypertension, diabetes, dyslipidemia and smoking habit were 25%, 2%, 20% and 31%, respectively. A family history of cardiovascular disease was present in 26% of the population. A previous history of myocarditis was present in four patients, all in the group without events at follow-up (*p* = 0.506). Both groups were homogeneous as regards age, sex, body mass index and rates of cardiovascular risk factors (*p* > 0.05). All patients were in New York Heart Association (NYHA) Class I, as HF at admission represented an exclusion criterion.

### 3.2. Biochemical Findings

A moderate increase in median C-reactive protein levels was observed (1.96 mg/dL, interquartile interval 0.62–5.42) without significant difference between groups (*p* = 0.775). This cohort of patients with events at follow-up also showed a higher relative neutrophilia (76 ± 7% vs. 61 ± 11%, *p* = 0.014), although the total count of white blood cells was homogeneous between groups. Similarly, no differences in serum creatinine levels or in troponin has emerged.

### 3.3. Baseline Echocardiographic Findings

Mean LVEF at admission was 57 ± 3%, with similar values between the two groups *(p* = 0.459). Only two patients showed a mild left ventricular dilation, while the mean LV end-diastolic diameter was 50 ± 4 mm; eight patients showed an increase of right ventricular end-diastolic diameter (population mean 32 ± 3 mm). Both indices were comparable in patients with and without events, as well as in the estimation of systolic pulmonary artery pressure. A total of 23 patients showed LV wall motion abnormalities, but the wall motion score index did not show statistically significant differences between the groups. No patient in the event group presented anterior wall hypokinesia.

### 3.4. Cardiac Magnetic Resonance Findings

CMR confirmed a mild LV dilation in only three patients, all males (indexed LV end-diastolic volume ≥ 105 mL/m^2^), and only one had an event at follow-up. Among them, only one patient had a slight reduction in LVEF at admission echocardiography that was not confirmed once CMR was performed. That patient did not meet the endpoint at follow-up. Only five males presented a slight decrease of LVEF at CMR (LVEF 50–55%), while mean CMR LVEF was 62 ± 5% with no difference between groups (*p* = 0.666). Interestingly, 15 patients showed a mild decrease in right ventricular EF, and four of them had events at follow-up. All patients presented normal RV dimensions.

**Figure 2:**
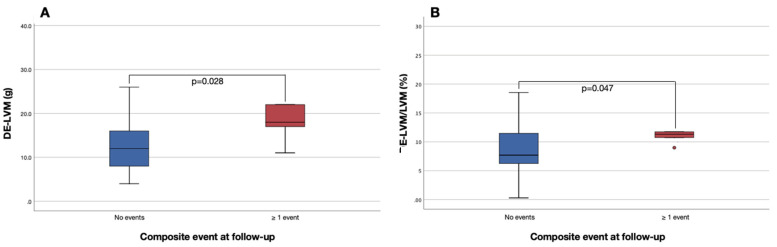
Box-plots for (**A**) left ventricular mass with delayed enhancement (DE-LVM) in patients who meet or do not meet the primary endpoint; (**B**) percentage of DE-LVM to the total left ventricular mass in patients who meet or do not meet the primary endpoint. DE, delayed enhancement; LVM, left ventricular mass.

Regional LV wall motion abnormalities were comparable between those with and without events, as were regional distribution of edema and LGE (all *p* > 0.05). Myocardial edema was found in 30 patients (49%) and LGE in 57 (93%). Despite similar distribution, the total amount of DE-LVM was statistically higher in patients with events at follow-up (18 (14–29.5) vs. 12 (8–16) g, *p* = 0.028; see Figure 2). The percentage of DE-LVM to the LVM also significantly differed between the two groups (patients with events: 11.3 (9.9–19.9); patients without events: 7.7 (6.2–11.6); *p* = 0.047).

### 3.5. Events at Follow-up

During the mean follow-up of 4.8 years for the whole population, we registered seven events: two patients died and five were hospitalized because of acute decompensated HF. Among the latter, only one patient presented a mildly decreased LVEF when decompensated at follow-up, while four presented with acute HF with preserved ejection fraction.

**Figure 3:**
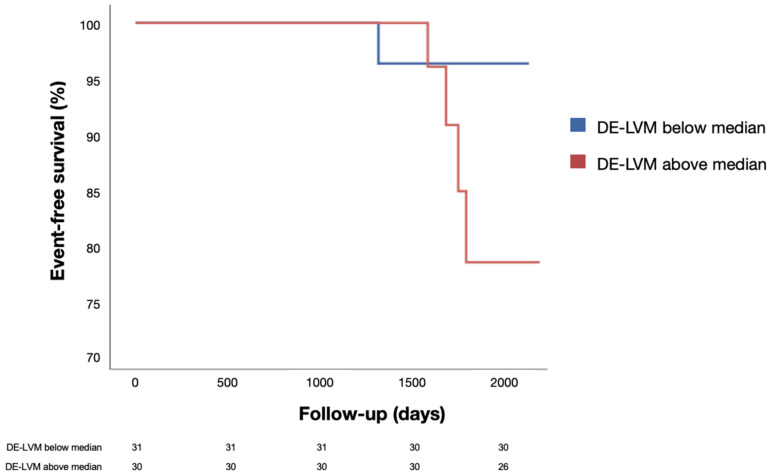
Kaplan–Meier survival curves for patients with left ventricular mass with delayed enhancement above (red) or below (blue) median. Numbers at risk are reported in lower part. DE, delayed enhancement; LVM, left ventricular mass.

At univariate Cox analysis, DE-LVM could predict the composite endpoint (*p* = 0.022; see Table 2 for univariate Cox analysis), as did the percentage of DE-LVM to LVM (*p* = 0.018). Furthermore, after splitting the population according to DE-LVM median value (see Table 3 for a comparison in baseline characteristics between the two groups), cumulative survival analysis by Kaplan–Meier showed a clear but not statistically significant divergence between 66-month curves (Figure 3, log rank 0.141). At follow-up visit at our outpatient clinic, three patients presented a moderate decrease in LVEF; interestingly, none of them had events at follow-up.

## 4. Discussion

In this study we evaluated a selected cohort of patients with AMpEF, whose management and prognosis are still challenging. Although simplistic, a classification depending on left ventricular ejection fraction (LVEF) helps in addressing clinical goals and investments towards a better comprehension of disease, especially as regards mid-to-long term management of myocarditis both with reduced and with preserved ejection fraction. The main result of the present study is that the amount—and not merely the presence or absence—of DE-LVM can predict the composite endpoint of all-cause mortality and hospitalization for HF in subjects presenting with AMpEF.

Although preserved systolic function is a good predictor of survival in many cases of heart disease, the presence of myocardial scar is generally associated with increased risk for adverse cardiovascular events (especially arrhythmias), even in patients with preserved systolic function [17]. Whether this applies to AMpEF still remains to be properly clarified.

There are contrasting data in the literature about the importance of CMR findings in patients with AMpEF. In 2014, Schumm et al. [18] evaluated 405 patients with suspected acute myocarditis and found that patients with abnormalities at CMR had a worse prognosis compared to patients without CMR findings. Normal CMR was defined as normal left ventricular volumes and normal LVEF in the absence of LGE. On the other hand, Sanguineti et al. [19] followed up 203 patients with preserved, mildly reduced or reduced LVEF and a CMR-based diagnosis of acute myocarditis for an average of 18.9 months. The authors observed that the presence and extent of myocardial edema and the extent of LGE were not predictive of the outcome for these patients, and an impaired LVEF at the index examination was the only independent CMR predictor of an adverse clinical outcome.

More recently, Aquaro et al. [7] in the ITAMY study demonstrated that among 386 patients with AMpEF and different patterns of LGE, the antero-septal phenotype was associated with a worse prognosis compared to the others and to patients without LGE. In particular, LGE in the mid-wall layer of the anteroseptal segments of patients with AMpEF was associated with a higher incidence of cardiac death, proper implantable cardioverter-defibrillator shock, resuscitated cardiac arrest, and hospitalization for HF at a median follow-up of 1572 days, as compared with other presentations. The extent of LGE was not different in patients with and without cardiac events. These data were further included in a meta-regression analysis by Georgiopoulos et al., who concluded that LGE presence and anteroseptal localization at baseline CMR are both independent prognostic markers in acute myocarditis. Anteroseptal localization had prognostic value irrespective of LVEF [6].

Despite the small sample size and being a single-center analysis, with our study we added a new datum in the comprehension of AMpEF: not only the presence of LGE, but also its amount is predictive of a worse outcome (*p* = 0.022). This was further confirmed when evaluating the percentage of DE-LVM to the LVM (*p* = 0.018). It might mean that also for relatively small fibrotic involvement that does not impair LVEF, even small differences in the percentage of left ventricular myocardium with LGE may affect outcome. In our population of AMpEF, no specific localization could predict the composite endpoint in our cohort. This finding is actually in discordance with results from the ITAMY study, but the small number of patients enrolled may explain this bias. Data from larger populations are awaited in order to clarify these different results.

Both groups of our study (patients with and without events at follow-up) were homogenous regarding clinical features, but AMpEF patients with events had higher levels of circulating neutrophils at admission. This may reflect a higher degree of inflammation, even if C-reactive protein levels were not significantly different between groups and routine assay of interleukins was not performed. In the ITAMY study [7], patients with anteroseptal localization had a higher troponin spillover, but C-reactive protein levels were counter-intuitively lower. From a pathophysiologic standpoint, the likely higher inflammatory response may explain the larger DE-LVM of patients with events at CMR. Indeed, a higher degree of myocardial inflammation in the acute phase may translate into a higher burden of fibrosis during the following healing phase. This, in turn, could explain the capability of DE-LVM to predict death and hospitalization for HF in our cohort. We also noticed that common risk factors for the development of HF with preserved ejection fraction did not differ between the two groups. In particular, patients with events at follow-up were not older or more hypertensive than those without events, and their medium BMI was comparable. Hence, risk factors that lead to acute decompensation still remain to be identified when considering the AMpEF phenotype as compared to others for HF with LVEF > 50%.

These findings might have several consequences, although derived from a single-center, retrospective analysis. First of all, CMR should be performed in all patients with AMpEF for different purposes: making diagnosis, confirming preliminary echocardiographic data of preserved LVEF and quantifying the extent of myocardial fibrosis. Our results may also reflect the need for a closer follow-up in order to re-assess myocardial function and arrhythmic burden. Moreover, a higher degree of fibrosis would require an early therapeutic approach that is still not available for this population. Randomized clinical trials, investigating the antagonism of both neurohormonal activity and directly fibrosis, are strongly required. As in HF, fibrosis is emerging not only as merely the consequence of myocardial insult, but also as a primary therapeutic target, and AMpEF should be no exception, especially if a large fibrotic process is detected.

### Limitations

This study has some limitations. First of all, this is a retrospective, single-center analysis on a small population of patients with AMpEF. We did not perform endomyocardial biopsy and the diagnosis was made by the summation of clinical and CMR findings; nonetheless, in clinical practice endomyocardial biopsy is rarely performed in patients with AMpEF because of disproportional risk-to-benefit ratio [20]. Furthermore, when compared with endomyocardial biopsy, CMR has been demonstrated to be very accurate for the detection of myocardial damage in acute myocarditis, whereas it was less sensitive for the diagnosis of chronic myocarditis. We also did not perform T1 and T2 mapping in our population; unfortunately, these two techniques were not available in our CMR scanner at the time of examination.

## 5. Conclusions

AMpEF is a subpopulation of patients currently having no evidence-based management. In our study, DE-LVM and the percentage of DE-LVM to LVM were the only predictors at long-term follow-up of the composite endpoint of all-cause mortality and hospitalization for HF in subjects presenting with AMpEF at baseline, according to a univariate analysis. These data confirm that not only the presence of LGE, but also the amount of DE-LVM, may have important prognostic roles also in AMpEF, as already demonstrated in other cardiomyopathies.

## Figures and Tables

**Table 1:** Descriptive analysis of our population of patients with acute myocarditis and preserved ejection fraction, divided according to cardiovascular (CV) events at follow-up. Significant *p* values are reported in bold.

	Whole Population (*n* = 61)	No CV Events (*n* = 54)	Composite CV Event (*n* = 7)	*p* Value
Baseline features				
Age (years)	39 ± 12	38 ± 12	44 ± 9	0.175
Sex (male)	49 (80)	42 (78)	7 (100)	0.327
Hypertension, *n* (%)	15 (25)	14 (26)	1 (14)	0.588
Dyslipidemia, *n* (%)	13 (21)	11 (20)	1 (14)	0.670
Diabetes, *n* (%)	1 (2)	0 (0)	1 (14)	0.130
Smoking habit, *n* (%)	19 (31)	17 (31)	2 (29)	0.661
Body mass index (kg/m^2^)	25.2 ± 3.6	25.3 ± 3.8	24.6 ± 1.2	0.702
Prior myocarditis, *n* (%)	4 (7)	4 (7)	0 (0)	0.506
Laboratory findings				
C reactive protein (mg/dL)	1.96 (0.62–5.42)	1.96 (0.62–5.87)	2.35 (0.80–3.40)	0.798
WBC (cells/mmc)	8792 ± 2681	8824 ± 2766	8530 ± 2137	0.813
Neutrophils (%)	63 ± 11	61 ± 11	76 ± 7	**0.014**
Hs-troponin (ng/L)	648 (251–1555)	710 (264–1583)	473 (90–1843)	0.677
Serum creatinine (mg/dL)	0.92 ± 0.12	0.91 ± 0.11	0.97 ± 0.15	0.452
GOT (UI/L)				
Baseline echocardiographic findings				
Baseline LVEF (%)	57 ± 3	57 ± 3	56 ± 2	0.459
IVS (mm)	9.9 ± 1.3	9.9 ± 1.3	10.0 ± 2.0	0.882
TAPSE (mm)	23 ± 4	23 ± 3	26 ± 7	**0.049**
sPAP (mmHg)	26 ± 5	25 ± 5	25 ± 5	0.798
Cardiac magnetic resonance findings				
Indexed LVEDV (mL/m^2^)	80 ± 14	80 ± 14	81 ± 15	0.970
Indexed LVM (g/m^2^)	75 ± 13	75 ± 13	74 ± 16	0.974
LVEF (%)	62 ± 5	62 ± 5	61 ± 8	0.666
Indexed RVEDV (mL/m^2^)	63 (58–75)	63 (58–75)	59 (53–68)	0.367
RVEF (%)	57 ± 8	57 ± 8	58 ± 8	0.876
Presence of myocardial edema, *n* (%)	30 (49)	26 (48)	4 (57)	0.707
IVS edema, *n* (%)	14 (23)	12 (22)	2 (29)	0.655
Lateral/inferolateral edema, *n* (%)	15 (25)	13 (24)	2 (29)	0.795
Inferior edema, *n* (%)	7 (12)	6 (11)	1 (14)	0.804
Anterior edema, *n* (%)	9 (15)	8 (15)	1 (14)	0.970
DE, *n* (%)	57 (93)	52 (96)	5 (71)	0.012
IVS DE, *n* (%)	16 (26)	14 (26)	2 (29)	0.881
Lateral/inferolateral DE, *n* (%)	44 (72)	40 (74)	4 (57)	0.386
Inferior DE, *n* (%)	14 (23)	11 (20)	3 (43)	0.335
Anterior DE, *n* (%)	7 (12)	7 (13)	0 (0)	0.586
Number of segments with DE, *n*	2.0 ± 1.7	2.0 ± 1.4	1.9 ± 1.5	0.813
DE-LVM (g)	12 (8–17)	12 (8–16)	18 (14–29.5)	**0.028**
DE-LVM/LVM, %	7.9 (6.4–11.7)	7.7 (6.2–11.6)	11.3 (9.9–19.9)	**0.047**
Follow-up				
Echocardiographic LVEF (%)	57 ± 4	57 ± 5	55 ± 2	0.261

DE, delayed enhancement; hs, high-sensitivity; GOT, glutamic-oxalacetic transaminase; IVS, interventricular septum; LVEDV, left ventricular end-diastolic volume; LVEF, left ventricular ejection fraction; LVM, left ventricular mass; RVEDV, right ventricular end-diastolic volume; RVEF, right ventricular ejection fraction; sPAP, systolic pulmonary artery pressure; TAPSE, tricuspid annular plane systolic excursion; WBC, white blood count.

**Table 2:** Univariate Cox analysis for the determination of predictors of the composite endpoint. Significant *p* values are reported in bold.

	B	HR	CI (95%)	*p* Value
Age	0.049	1.050	0.985–1.120	0.131
C reactive protein	−0.138	0.872	0.639–1.189	0.386
Neutrophils	0.010	1.010	0.962–1.061	0.677
Hs-troponin	0.045	1.045	0.979–1.028	0.860
sPAP	0.006	1.006	0.811–1.248	0.957
LVEF	−0.017	0.983	0.850–1.136	0.814
RVEF	0.010	1.010	0.909–1.122	0.856
IVS DE	−0.123	0.884	0.171–4.583	0.884
DE-LVM	0.122	1.130	1.017–1.256	**0.022**
DE-LVM/LVM	4.573	2.132	1.272–3.573	**0.018**

CI, confidence interval; DE, delayed enhancement; HR, hazard ratio; IVS, interventricular septum; LVEF, left ventricular ejection fraction; LVM, left ventricular mass; RVEF, right ventricular ejection fraction; sPAP, systolic pulmonary artery pressure.

**Table 3:** Descriptive analysis of our population of patients with acute myocarditis and preserved ejection fraction, divided into two groups (above or below median value of left ventricular mass with delayed enhancement). Significant p values are reported in bold.

	Whole Population (*n* = 61)	DE-LVM below Median Value (*n* = 31)	DE-LVM above Median Value (*n* = 30)	*p* Value
Baseline features				
Age (years)	39 ± 12	38 ± 12	38 ± 10	0.175
Sex (male)	49 (80)	21 (68)	28 (93)	0.327
Hypertension, *n* (%)	15 (25)	9 (29)	6 (20)	0.592
Dyslipidemia, *n* (%)	13 (21)	7 (23)	6 (20)	0.856
Diabetes, *n* (%)	1 (2)	0	1 (3)	0.130
Smoking habit, *n* (%)	19 (31)	11 (35)	8 (27)	0.948
Body mass index (kg/m^2^)	25.2 ± 3.6	25.0 ± 3.8	25.5 ± 3.5	0.418
Prior myocarditis, *n* (%)	4 (7)	2 (6)	2 (7)	0.506
Laboratory findings				
C reactive protein (mg/dL)	1.96 (0.62–5.42)	1.82 (0.45–4.35)	2.70 (0.67–7.00)	0.775
WBC (cells/mmc)	8792 ± 2681	8794 ± 2823	8738 ± 2753	0.813
Neutrophils (%)	63 ± 11	59 ± 11	64 ± 11	**0.014**
Hs-troponin (ng/L)	648 (251–1555)	396 (195–1055)	720 (355–1915)	0.669
Serum creatinine (mg/dL)	0.92 ± 0.12	0.91 ± 0.11	0.93 ± 0.13	0.452
GOT (UI/L)	27 (18–54)	20 (16–29)	40 (23–71)	0.296
Baseline echocardiographic findings				
Baseline LVEF (%)	57 ± 3	57 ± 3	57 ± 3	0.459
IVS (mm)	9.9 ± 1.3	9.4 ± 1.1	10.3 ± 1.4	0.882
TAPSE (mm)	23 ± 4	24 ± 4	23 ± 4	**0.049**
sPAP (mmHg)	26 ± 5	25 ± 3	26 ± 6	0.798
Cardiac magnetic resonance findings				
Indexed LVEDV (mL/m^2^)	80 ± 14	78 ± 11	83 ± 15	0.970
Indexed LVM (g/m^2^)	75 ± 13	69 ± 9	81 ± 14	0.974
LVEF (%)	62 ± 5	63 ± 5	61 ± 5	0.666
Indexed RVEDV (mL/m^2^)	63 (58–75)	62 (57–68)	66 (60–75)	0.321
RVEF (%)	57 ± 8	57 ± 9	57 ± 7	0.876
Presence of myocardial edema, *n* (%)	30 (49)	11 (35)	19 (63)	0.707
IVS edema, *n* (%)	14 (23)	5 (16)	9 (30)	0.655
Lateral/inferolateral edema, *n* (%)	15 (25)	4 (13)	11 (37)	0.795
Inferior edema, *n* (%)	7 (12)	3 (10)	4 (13)	0.804
Anterior edema, *n* (%)	9 (15)	5 (16)	4 (13)	0.971
DE, *n* (%)	57 (93)	27 (87)	30 (100)	0.061
IVS DE, *n* (%)	16 (26)	5 (16)	11 (37)	0.882
Lateral/inferolateral DE, *n* (%)	44 (72)	22 (70)	22 (73)	0.386
Inferior DE, *n* (%)	14 (23)	6 (19)	8 (27)	0.335
Anterior DE, *n* (%)	7 (12)	3 (10)	4 (13)	0.586
Number of segments with DE, *n*	2.0 ± 1.7	1.4 ± 0.6	2.7 ± 1.4	0.813
DE-LVM/LVM, %	7.9 (6.4–11.7)	6.3 (4.9–7.7)	11.3 (8.1–12.8)	**<0.001**
Follow-up				
Echocardiographic LVEF (%)	57 ± 4	58 ± 3	57 ± 6	0.070

DE, delayed enhancement; hs, high-sensitivity; GOT, glutamic-oxalacetic transaminase; IVS, interventricular septum; LVEDV, left ventricular end-diastolic volume; LVEF, left ventricular ejection fraction; LVM, left ventricular mass; RVEDV, right ventricular end-diastolic volume; RVEF, right ventricular ejection fraction; sPAP, systolic pulmonary artery pressure; TAPSE, tricuspid annular plane systolic excursion; WBC, white blood count.

## Data Availability

The datasets generated during and/or analyzed during the current study are available from the corresponding author on reasonable request.

## Abbreviations

AMpEF	acute myocarditis with preserved ejection fraction
CMR	cardiac magnetic resonance
DE-LVM	delayed enhancement-left ventricular mass
HF	heart failure
LGE	late gadolinium enhancement
LV	left ventricular
LVEF	left ventricular ejection fraction
NYHA	New York Heart Association
TAPSE	tricuspid annular plane systolic excursion
